# Stannous fluoride forms aggregates between outer and inner membranes leading to membrane rupture of *Porphyromonas gingivalis* and *Prevotella pallens*

**DOI:** 10.3389/froh.2024.1427008

**Published:** 2024-06-26

**Authors:** Sancai Xie, Vighter Iberi, Ying Boissy, Cheryl S. Tansky, Tom Huggins, Niranjan Ramji, Aaron R. Biesbrock

**Affiliations:** ^1^ Discovery & Innovation Platforms, The Procter & Gamble Company, Mason, OH, United States; ^2^ Global Oral Care R&D, The Procter & Gamble Company, Mason, OH, United States

**Keywords:** stannous fluoride, antibacterial agents, transmission electronic microscopy, gram-negative bacteria, mode of action

## Abstract

**Objective:**

Stannous has been shown to bind to free lipopolysaccharides, thus preventing them from binding to TLR receptors. This study was undertaken to determine the histomorphological mechanism of stannous binding to anaerobic bacteria.

**Methods:**

Two bacteria associated with gingivitis and advanced periodontal disease, *Porphyromonas gingivalis* (*P. gingivalis*) and *Prevotella pallens (P. pallens)*, were cultured in 25–1,000 μM of stannous fluoride and stannous chloride for 48 h. The growth rate was estimated using absorbance OD600. Bacterial cells were then fixed and processed for transmission electron microscopy (TEM) analysis.

**Results:**

Stannous fluoride inhibited proliferation of both *P. gingivalis* and *P. pallens* in a dose-dependent manner. There was a statistically significant suppression of the growth curve starting at 100 μM for *P. pallens* (*P* = 0.050) and 200 μM for *P. gingivalis* (*P* = 0.039). TEM analysis revealed a thick layer of polysaccharides (19.8 nm) in *P. gingivalis*. The outer and inner membranes were clearly visible with low electron densities in both bacteria. Stannous diffused into bacterial membranes and formed precipitates in the areas spanning outer and inner membranes and below inner membranes. Precipitates varied in size ranging from 46.4 to 84.5 nm in length, and 18.4 to 35.9 nm in width. The membranes were disintegrated in the region where stannous formed precipitates. Cytosolic contents were leaked out, and in several cases, small vesicles were formed. Stannous precipitates were more abundant in numbers and larger in size in bacteria treated with high concentrations (100–300 μM) than in low concentrations (25–50 μM) of stannous fluoride. Furthermore, most of the bacteria were disintegrated in the groups treated with 100–300 μM stannous fluoride. At low concentrations (25 μM), stannous fluoride formed complexes primarily around outer membranes, to which lipopolysaccharides are anchored. Stannous chloride results showed similar trends, but it was less potent than stannous fluoride.

**Conclusion:**

Stannous fluoride can penetrate bacteria, bind to the constituents of the membrane and form precipitates between outer and inner membranes and beneath inner membranes. These large precipitates damaged the integrity of membranes and allowed cytosolic contents to be leaked out. Stannous complexes formed at the outer membranes, even at low concentrations (25 μM).

## 1 Introduction

Stannous fluoride is formulated in oral care products for the control of both dental caries and gingival inflammation (1, 2). The clinical efficacy of stannous fluoride dentifrice for gingivitis reduction is imparted primarily through antimicrobial actions on bacterial flora in the mouth. Stannous fluoride reduces both the quantity of dental plaque (3) and modulates the metabolic activity of dental plaque (4, 5). Complementing these antimicrobial properties, stannous fluoride has been shown to have a strong affinity to directly bind bacterial endotoxins, such as lipopolysaccharides (LPS) (6–8). This reactivity with lipopolysaccharides suppresses the promotion of Toll-like receptors, which activate signal pathways provoking the onset and progression of gingival inflammation (6–8). Cumulatively, these activities help explain the proven clinical efficacy of stannous fluoride formulations in improving gingival health (9). A recent meta-analysis of 18 randomized controlled gingivitis clinical studies in 2,890 subjects reported a mean reduction of 51% of bleeding sites in subjects using stannous fluoride dentifrice vs. a negative control (sodium fluoride or sodium monofluorophosphate) dentifrice for periods of 3 months or less (10). Furthermore, subjects with localized to generalized gingivitis at baseline had 3.7 times better odds of shifting from gingivitis case type (≥10% bleeding sites) to generally healthy case type (<10% bleeding sites) using stannous fluoride vs. negative control dentifrices (10). This research demonstrates that the cation of the stannous fluoride molecule provides specific antimicrobial action beyond that of fluoride alone. Other stannous fluoride formulations have been shown to be effective against gingivitis (11). The formulation chosen for this investigation demonstrated the greatest gingival health benefit in a clinical evaluation of three different stannous fluoride dentifrices (12).

The importance of *Porphyromonas gingivalis* (*P. gingivalis)* and *Prevotella* species in dental plaque dysbiosis leading to gingival inflammation and the initiation of gingivitis has been well characterized (13–16). A randomized controlled experimental gingivitis study in 91 subjects confirmed that during a 21-day period of cessation of oral hygiene, the unrestricted growth of the dental biofilm led to a dysbiotic microflora demonstrating an increasing overabundance of *Porphyromonas* and *Prevotella* species (13). This is consistent with previous studies demonstrating associations between *P. gingivalis* and *Prevotella* species and the presence of gum disease. *P. gingivalis* and *Prevotella pallens* (*P. pallens*, formerly *P. denticola*) were found to be differentially overabundant in chronic periodontitis vs. healthy and demonstrated increasing overabundance during experimental gingivitis (14). *P. pallens* is closely related to *Prevotella intermedia* (*P. intermedia*) and belongs to the same *Prevotella* family which includes *Prevotella nigrescens* and *Prevotella aurantica* (15). *P. intermedia* has been reported to increase during the onset of pregnancy gingivitis (16). In a separate experimental gingivitis clinical study, the relative abundance of *Prevotella oulorum* was found to be significantly positively correlated to bleeding on probing scores that are a clinical measure of gingivitis (14).

The direct reactivity of stannous fluoride with bacteria is important to all aspects of its clinical efficacy. In previous studies stannous fluoride was shown to deposit within *Streptococcus mutans (S. mutans),* a Gram-positive bacteria associated with dental caries (17). The bacteria associated with gingival inflammation include Gram-negative anaerobic bacteria that proliferate in dental plaque biofilms at and below the gumline (18). While the membrane structure of *S. mutans* include peptidoglycan (19), the structure of Gram-negative periodontal pathogens is different, being comprised of inner and outer cell membranes containing enriched lipopolysaccharides, membrane proteins as well as bilayers of phospholipids (20). Because stannous fluoride so strongly binds with lipopolysaccharides, we questioned whether this reactivity might contribute to bactericidal actions of stannous fluoride against periodontal pathogens. Recent research has reaffirmed that stannous ions from stannous fluoride penetrate into *in situ* biofilm and that stannous accumulation occurs primarily within *P. gingivalis* (21, 22). Here, we conducted a study using transmission electron microscopy (TEM) to histomorphologically visualize the mechanism of stannous fluoride reactivity and bactericidal activity on *P. gingivalis* and *P. pallens*.

## 2 Materials and methods

Stannous fluoride, stannous chloride and endotoxin-free ultra pure water were purchased from Sigma (St. Louis, MO). MTGE-anaerobic enrichment broth was purchased from Anaerobe Systems (Morgan Hill, CA). MTGE-anaerobic enrichment broth and pure water were acclimated under an anaerobic conditions to remove residual oxygen. Fresh solutions of stannous fluoride and stannous chloride were prepared right before each experiment to prevent stannous oxidation to stannic, which is not active in inhibiting bacterial growth.

### 2.1 Bacterial culture and treatment

In preliminary experiments to define the experimental dose response, stannous fluoride lysed almost all Gram-negative bacteria at 1 mM. Thus, a dose-curve study was run between 0 and 300 µM to find the concentrations of stannous fluoride and stannous chloride that inhibit bacterial growth and kill bacteria. Two experiments were conducted with three replicates per experiment for each bacteria. The culture conditions were optimized to visualize morphological changes in *P. gingivalis* and *P. pallens* in a range of stannous fluoride doses close to its minimum inhibitory concentration (MIC) (23), which was 200 µM under our culture conditions. Bacterial growth was slow in the MTGE medium. *P. pallens* ATCC 700821 and *P. gingivalis* ATCC 33277 were grown on MTGE anaerobic enrichment broth under anaerobic conditions with a N_2_, CO_2_, H_2_ gas mixture ratio of 80:10:10 at 37°C for 48 h in a 5 ml Falcon tube. The culture started with an inoculation of an overnight growth culture at an optical density (OD600) of 0.1. Stannous solutions were first made in sterile water and diluted into MTGE-anaerobic enrichment broth. Individual cultures were then harvested at 48 h (to ensure culture growth to stationary phase) and OD600 was measured using Molecular Devices SpectraMax M5 Multilabel Microplate Reader (San Jose, CA). Results were analyzed using package ggpubr of R program in RStudio. The input result included only the experiment, dose of stannous and mean OD600 of each experiment. One way Analysis of Variance (ANOVA) was used to determine the treatment effect of stannous fluoride and stannous chloride. Individual concentrations were compared using pairwise *t*-test.

### 2.2 TEM sample preparation and imaging

Bacterial cultures were centrifuged to collect the bacteria from the 48 h culture step, and the bacterial pellets were placed in a fixative solution (2% glutaraldehyde in PBS buffer) immediately, and stored at 4 ℃. The fixed samples were rinsed in 1× PBS buffer 3 times and post-fixed with 1% OsO_4_ in PBS buffer overnight at 4 ℃. The samples were then dehydrated with a series of acetone concentrations (50%, 70%, 85%, 90%, 100%) for 3 h at room temperature, and infiltrated by gradually increasing the concentration of Epon 812 epoxy resin with acetone (30%, 50%, 75%, 100%) at room temperature. The completely infiltrated samples were embedded in 100% Epon 812 epoxy resin (Electron Microscopy Sciences, Hatfield, PA) and cured overnight at 65℃. Each sample block was trimmed and sectioned using a Leica UC6 ultramicrotome (Leica Microsystems, Deerfield, IL) with a Diatome ultracut 45° diamond knife. Roughly 70 nm thick sections were collected, placed on 200 mesh copper grid with formvar, and post-stained with uranyl acetate for 30 min and lead citrate for 10 min. Transmission electron microscopy (TEM) was performed using a Hitachi S5200 STEM (Hitachi High-Tech, Hillsboro, OR) for high resolution imaging analysis at 30 kV and with Bruker Quantax EDS detector (Bruker, Madison, WI) for elemental analysis.

## 3 Results

### 3.1 Inhibition of bacterial growth by stannous fluoride

**Figure 1:**
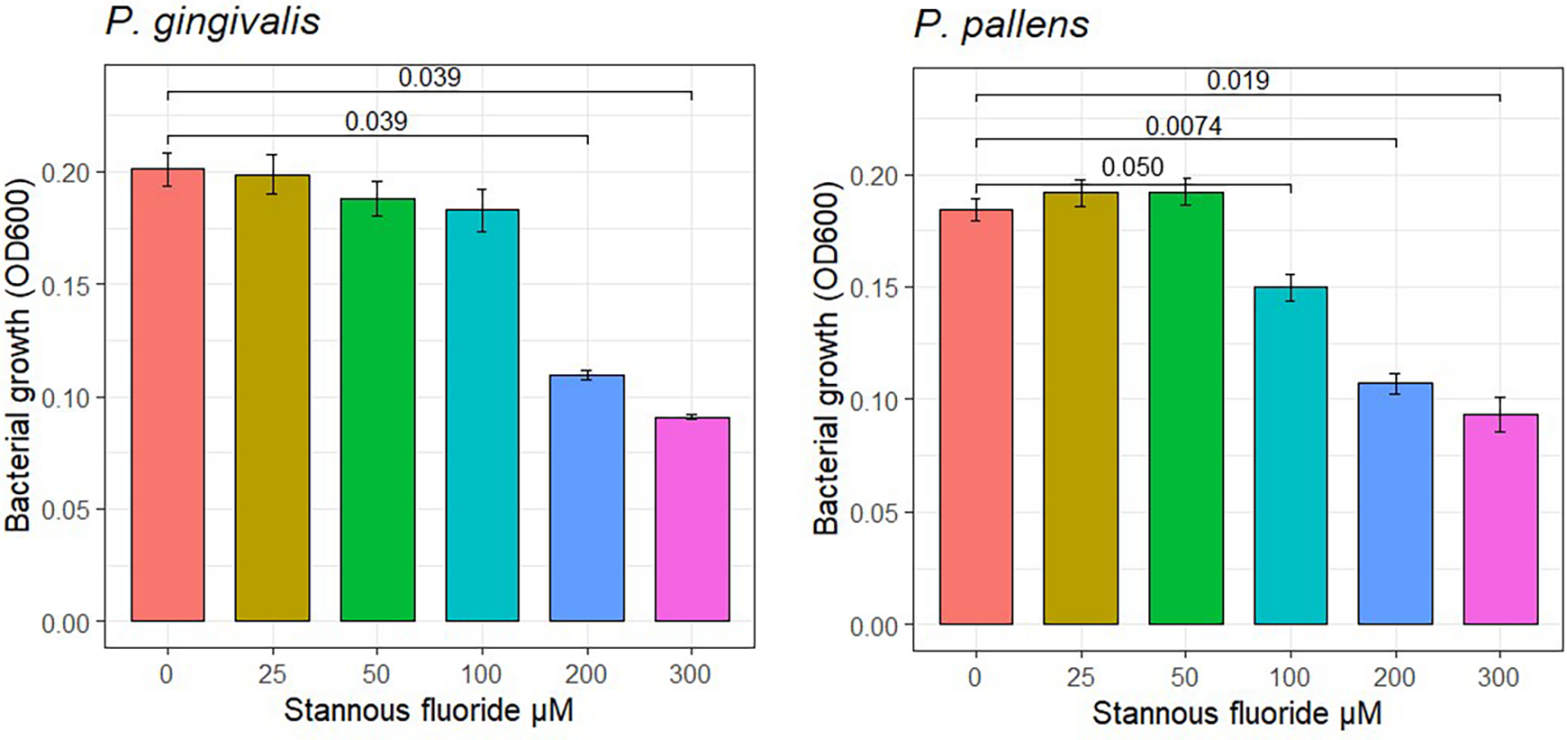
Effects of stannous fluoride on bacterial growth. One way Analysis of Variance (ANOVA) was used to determine the treatment effect of stannous fluoride. Individual concentrations were compared using pairwise *t*-test. Mean values and standard errors are representative of 6 total observations from two independent experiments (3 replicates in experiment). Cell growth was detected after culture for 48 h.

The effect of stannous fluoride on bacterial growth of *P. gingivalis* and *P. pallens* is shown in Figure 1. Stannous fluoride concentrations suppressed bacterial growth starting at 100 μM for *P. pallens* (*P* = 0.050) and 200 μM for *P. gingivalis* (*P* = 0.039). There was a positive dose response effect, with growth rates declining as stannous fluoride concentrations increased from 100 µM to 300 µM.

### 3.2 Ultrastructure of *P. gingivalis* and *P.* *pallens* grown in nutrient-enriched broth

**Figure 2:**
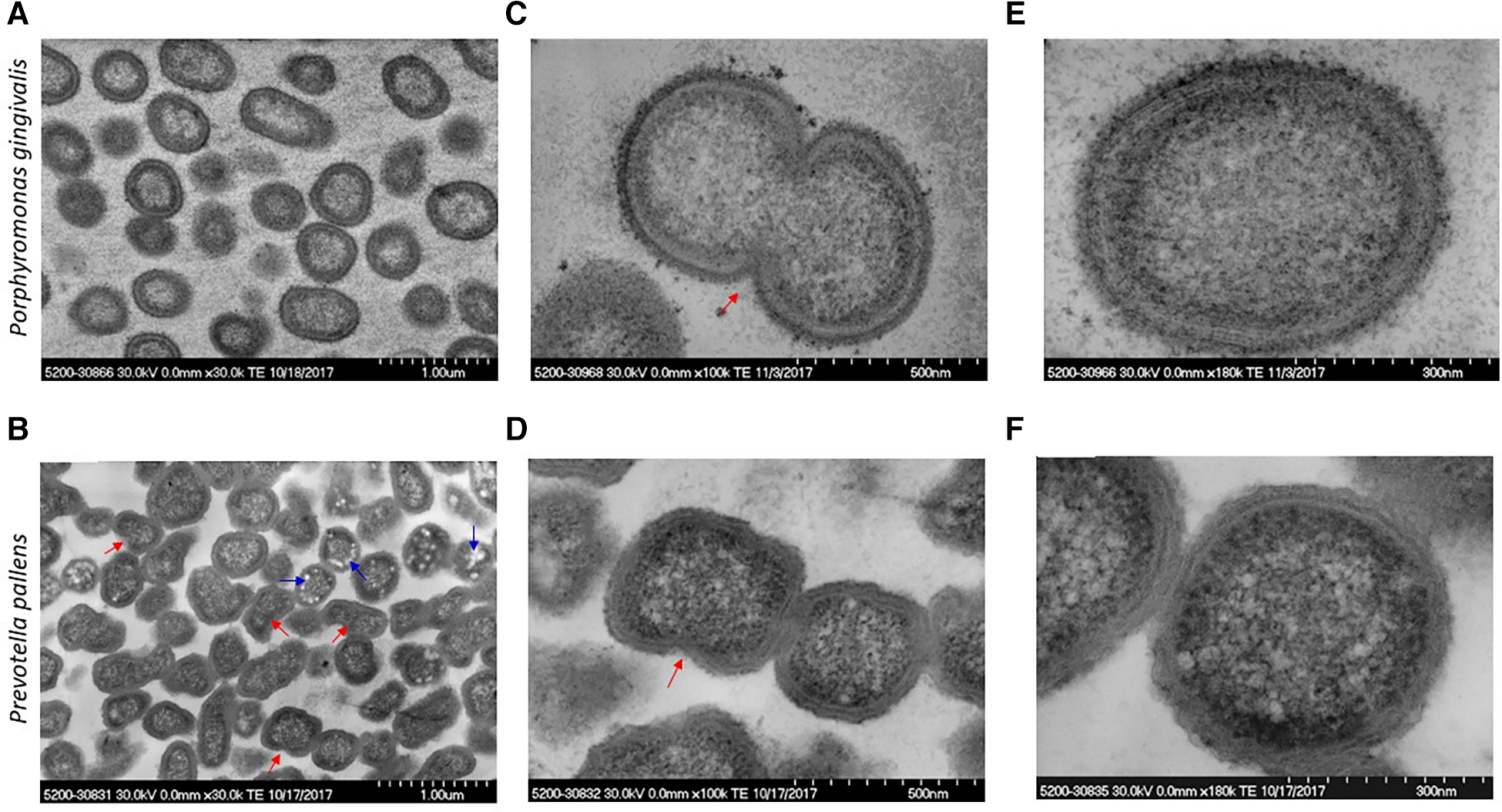
Micrographs of *P. gingivalis* and *P. pallens* in cross-section. Bacteria were grown in MTGE medium. Graphs (**A**,**C**,**E**) show the structure of *P. gingivalis*. Graphs (**B**,**D**,**F**) show the structure of *P. pallens*. Red arrows indicate cell division using binary fission. The blue arrows indicate electron-lucent granules.

The ultrastructure was compared between *P. gingivalis* and *P. pallens*. The structural features of *P. gingivalis* and *P.* *pallens* were revealed by TEM in Figure 2. These Gram-negative anaerobes exhibit characteristic cell membranes comprised of lipopolysaccharides (endotoxin) and phospholipids that are clearly observed (Figures 2E,F). Cellular contents were evenly distributed within the cytoplasmic membrane. Importantly, binary fission was visible in both *P. gingivalis* and *P. pallens* (red arrow, Figures 2B–D). In *P. pallens* (Figure 2B), some round bright vacuoles (blue arrows) were abundant, which are electron-lucent granules (24) The term “electron-lucent” refers to their appearance in the TEM image as loose lighter contrast aggregates. These vacuoles might be rich in lipids or other substances that do not provide significant image contrast (25).

**Figure 3:**
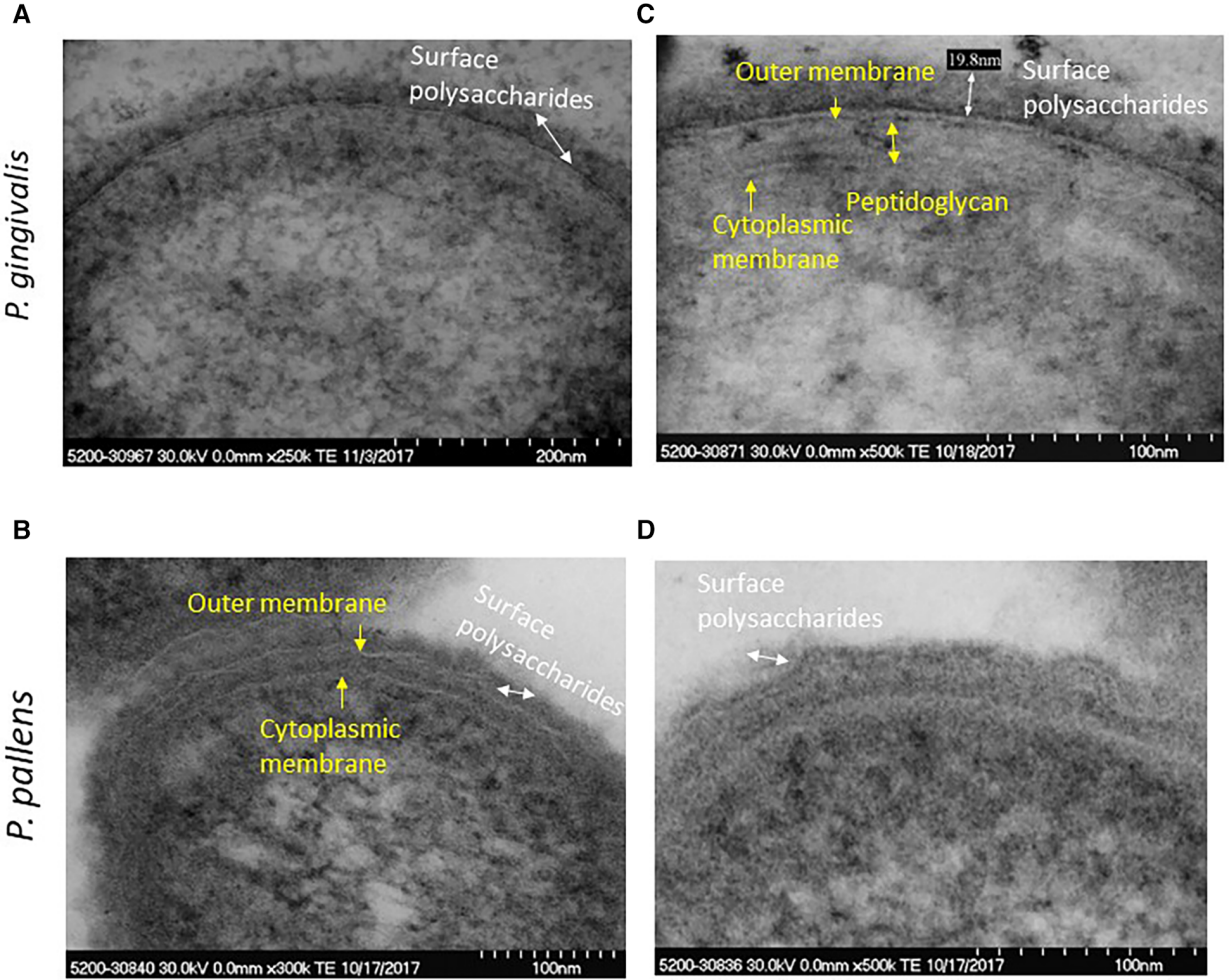
High resolution image of *P. gingivalis* and *P. pallens* cell membranes. (**A**,**C**) represent *P. gingivalis*. (**B**,**D**) represent *P. pallens*.

Supplementary Figure S1 shows morphological characteristics of *P. pallens* and *P. gingivalis*. The high-resolution view of *P. pallens* and *P. gingivalis* showed thick layers of peptidoglycans (Figures 3B,C). *P. gingivalis* showed both outer and cytoplasmic membranes (Figure 3C). A thick layer of surface polysaccharides was evident (19.8 nm, Figures 3A–D) which contains the major cell surface macromolecules, including capsular polysaccharide (or K-antigen), extracellular polysaccharides and LPS (26, 27). Those macromolecules on the surface of bacteria confer ultrastructural stability and form a defensive barrier against the host's immune system and environment stresses. Those surface macromolecules are highly virulent to gingival tissue and innate immune cells (25, 28).

### 3.3 Ultrastructure of *P. gingivalis* and *P. pallens* treated with stannous fluoride

**Figure 4:**
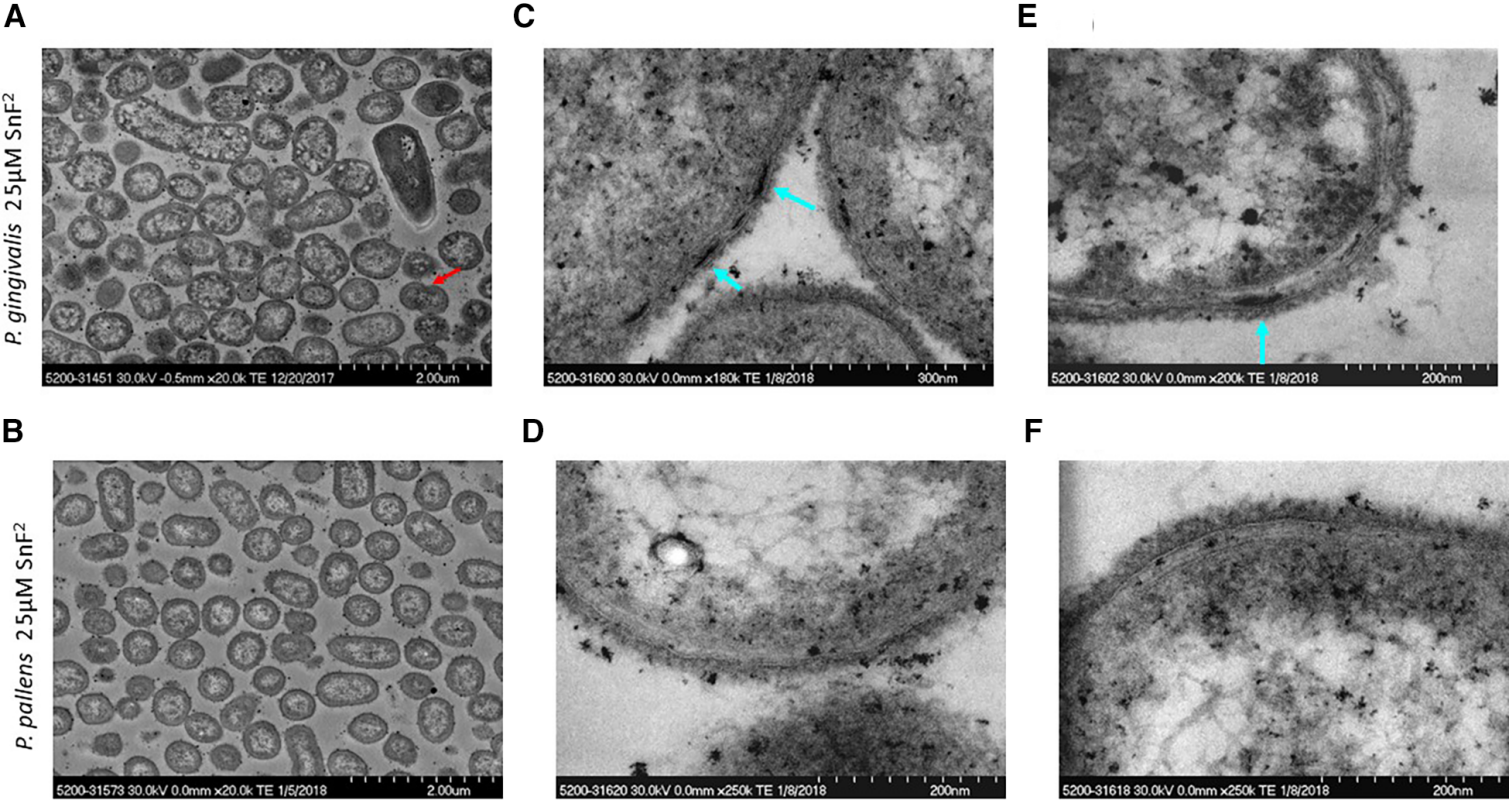
TEM micrographs of *P. gingivalis* and *P. pallens* treated with 25 µM stannous fluoride. (**A**,**C**,**E**) represent *P. gingivalis*. (**B**,**D**,**F**) represent *P. pallens*. Turquoise arrows indicate deposits of stannous aggregates and the red arrow indicates bacterial division.

Growth of both *P. gingivalis* and *P. pallens* was not suppressed by stannous fluoride at 25 µM. Bacterial division was evident as marked by a red arrow (Figure 4A). Cell membranes were visible, and cytoplasm was evenly distributed (Figure 4). Nonetheless, aggregate of stannous fluoride was formed on the outer membranes as indicated by the turquoise arrows in Figures 4C,E. The different concentrations of stannous fluoride resulting in aggregation vs. growth inhibition indicate a threshold effect is required to move from deposition to bactericidal activity. Electron-dense particles are also seen intracellularly in Figure 4. These particles originate from the post-fixation treatment with osmium tetroxide (Os signal) and the phosphate buffer saline (P and Cl signals) as shown in Supplementary Figure S2. The observed copper signal is not localized intracellularly and originates from the copper grid which was used as a support for the TEM sections.

**Figure 5:**
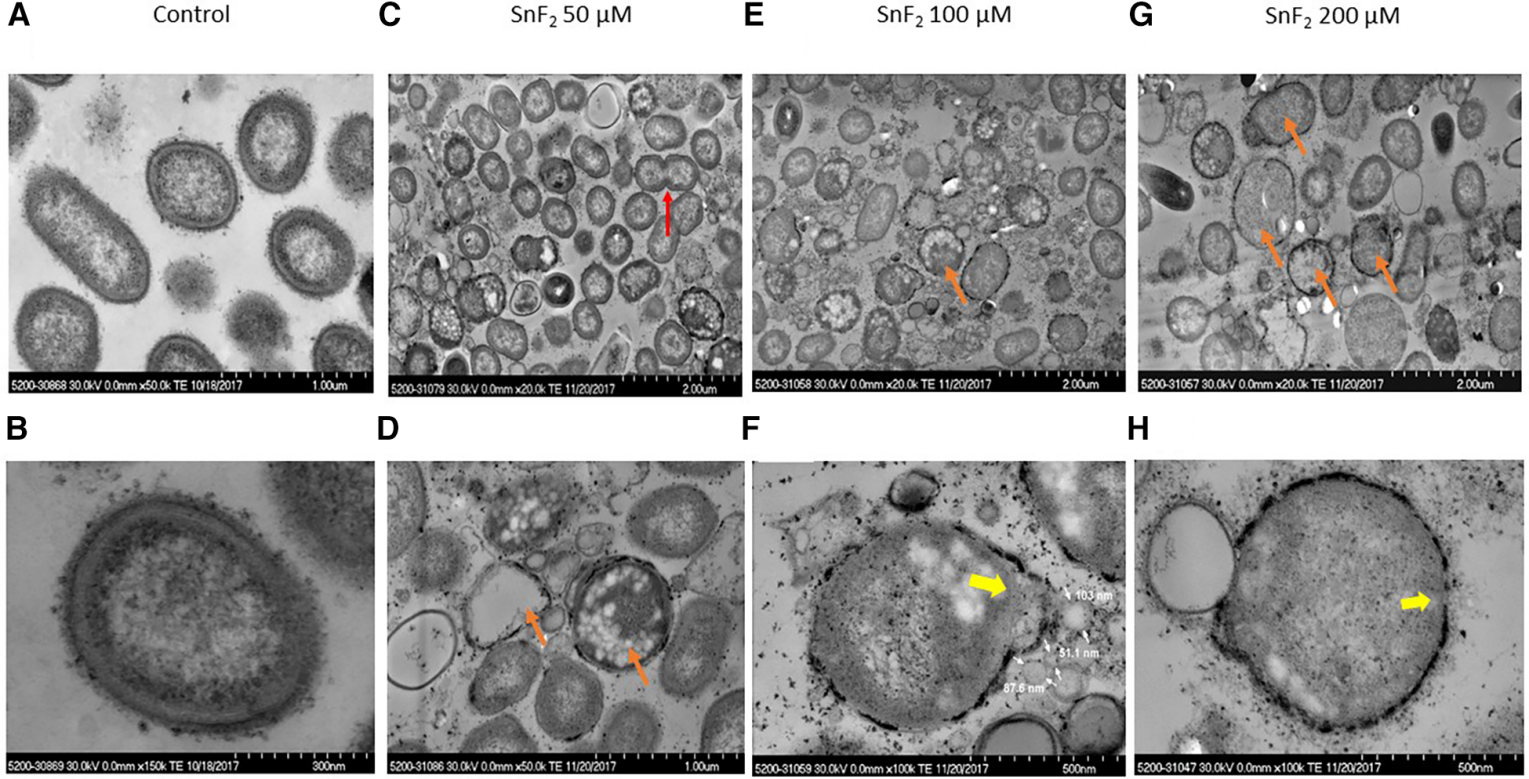
Observations of *P. gingivalis* treated with stannous fluoride. *P. gingivalis* was treated with stannous fluoride at 0 µM [control images (**A**,**B**)] 50 µM [images (**C**,**D**)], 100 µM (**E**,**F**), and 200 µM (**G**,**H**). Electron dense aggregates appeared on and inside cell membranes. Bacterial lysis was observed throughout. Some deposits were observed on the surface. The red arrow indicates bacterial division, orange arrows mark bacteria cloaked with electron-dense spots and partial or total loss of cellular contents, yellow arrows indicate explosive disruption of bacterial cell walls.

Stannous fluoride inhibited *P. gingivalis* growth at 200 µM of stannous fluoride and lysed the bacteria starting at concentrations of 100 µM (Figure 5). Bacterial division was observed as indicated by a red arrow at 50 µM of stannous fluoride (Figure 5C). Many bacteria were still full of cellular organelles at 50 µM of stannous fluoride, but not at 100–200 µM (Figures 5C,E). Stannous fluoride formed aggregates between and around the outer and cytoplasmic membranes (Figures 5D,F,H). As we reported previously, stannous fluoride bound LPS at the lipid A moiety of purified LPS. Lipid A of the LPS resides inside the outer leaflet of the outer membrane. The stannous aggregates disrupted bacterial cell walls and allowed the cellular contents and organelles to leak out as indicated by the yellow arrows (Figures 5F,H). Vesicles of varied sizes were formed in the disrupted site (Figure 5F, also Supplementary Figures 3A,B). These vesicles are formed in a process called explosive cell lysis (29). Stannous aggregates destroyed the integrity of bacterial membranes, which self-anneal to form explosive outer–inner membrane vesicles and explosive outer membrane vesicles. These vesicles contain cytoplasmic proteins, DNA and RNA. It is worth noting that explosive outer membrane vesicles are different from those mediated by outer membrane blebbing during bacterial growth.

Leakage of cellular organelles was often observed with stannous aggregates in the membranes of *P. gingivalis*. The bacterium, that is devoid of cellular organelles due to leakage, was cloaked in a ring of electron-dense spots, as marked by an orange arrow (Figures 5D,E,G) and by a yellow arrow (Figures 5F,H). Those electron-dense spots were stannous aggregates as confirmed by energy dispersive x-ray spectroscopy (EDS), which is described in Section 3.4.

**Figure 6:**
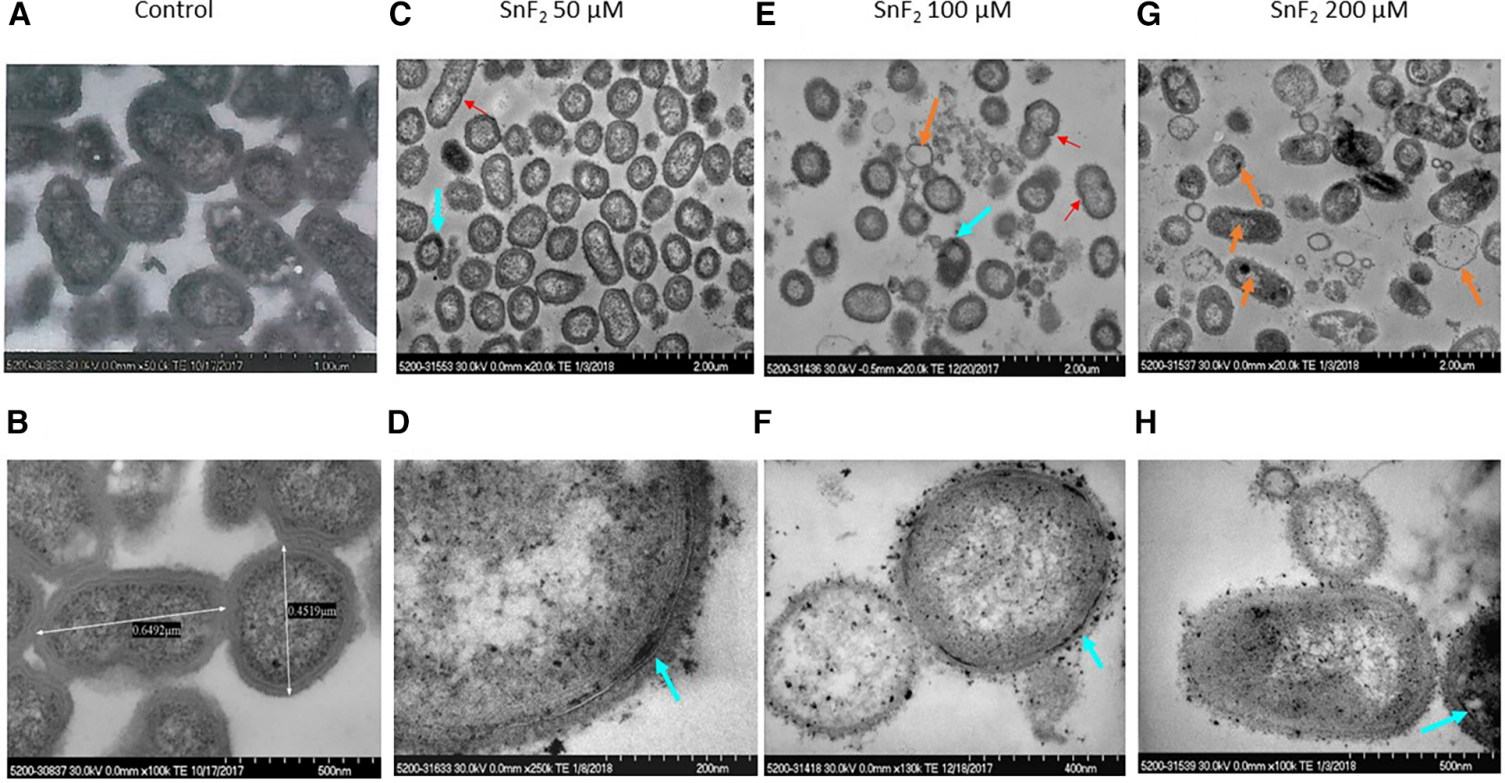
Observations of *P. pallens* treated with stannous fluoride. *P. pallens* was treated with stannous fluoride at 0 µM [control images (**A**,**B**)] 50 µM [images (**C**,**D**)], 100 µM (**E**,**F**), and 200 µM (**G**,**H**). Electron dense aggregates appeared on and inside cell membranes. Bacterial lysis was observed throughout. The red arrow indicates bacterial division, orange arrows indicate leakage of cellular contents, turquoise arrows indicate deposits of stannous aggregates.

Stannous fluoride did not inhibit *P. pallens* growth at 50 µM as measured by OD600 (Figure 1B). Bacterial division was visible at 50 µM and even 100 µM of stannous fluoride as indicated by red arrows (Figures 6C,E). Stannous fluoride formed aggregates within the outer and cytoplasmic membranes as indicated by the turquoise arrows (Figures 6C–F,H). These aggregates destabilized and damaged the bacterial cell walls. Consequently, cellular contents were emptied as indicated by the orange arrows (Figures 6E,G). It is worth noting that small vesicles were present right next to a bacterium, in which aggregates of stannous fluoride were visible in the membranes (turquoise arrows in Figures 6C,E).

**Figure 7:**
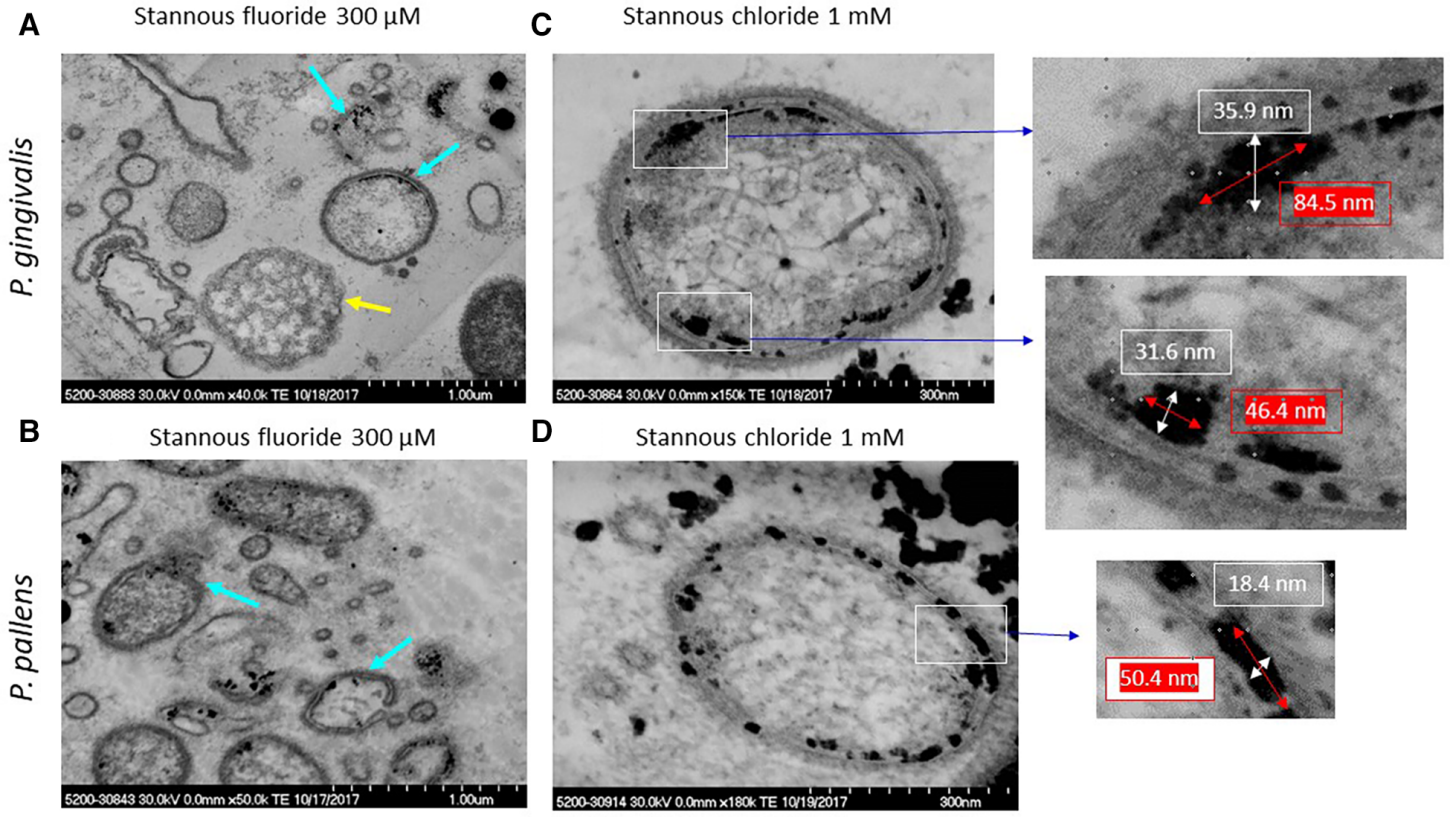
TEM micrographs of *P. gingivalis* and *P. pallens* treated with stannous fluoride and stannous chloride. (**A**,**C**) were *P. gingivalis*; (**B**,**D**) were *P. pallens*. Turquoise arrows indicate aggregates of stannous fluoride; a yellow arrow indicates a cellular remnant without visible membranes.

High concentrations of stannous fluoride (0.3 and 1 mM) lysed both *P. gingivalis* and *P. pallens* (Figures 7A,B). The cell contents leaked out, leaving a ring of cell membranes with stannous fluoride aggregates as indicated by turquoise arrows. Small vesicles were visible near those bacterial membrane rings. Some of the membrane fragments self-annealed to form those explosive vesicles. One bacterial remnant was observed in Figure 7A as indicated by a yellow arrow. The cellular contents were devoid of apparent outer and cytoplasmic membranes.

Similar cellular remnants were also observed in Supplementary Figures S3A,B. Bacteria were treated with toothpaste which contained both stannous fluoride and stannous chloride (Crest Pro-Health, Procter & Gamble) as described in Supplementary Methods. Stannous formed aggregates in the membranes treated with stannous fluoride solution as indicated by a turquoise arrow. Bacterial membranes rolled to form small vesicles.

Figures 7C,D show samples treated with 1 mM stannous chloride, at which concentration full bacterial cells were still present. Stannous chloride formed large aggregates in and around the membranes (Figures 7C,D). A lipid bilayer of cell membranes is typically about 5–10 nm thick. The aggregates were larger, ranging from 18.4 nm between the outer and cytoplasmic membranes and 35.9 nm around the membranes in thickness and 46.4–84.5 nm in length. These aggregates visibly disrupted the integrity of the membranes.

### 3.4 Confirmation of stannous in the aggregates

**Figure 8:**
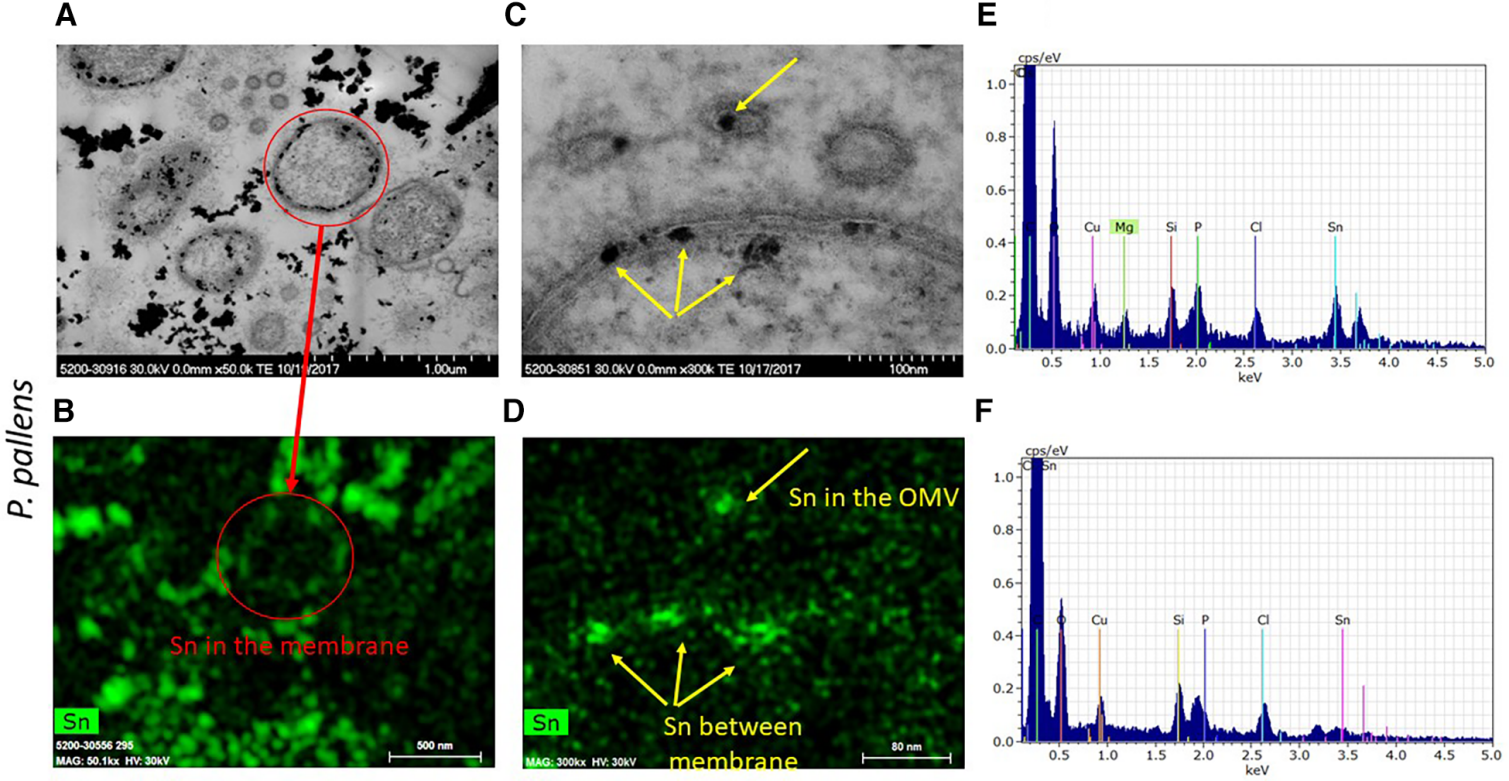
Micrographs of electron dense granules observed in *P. pallens* incubated with 1 mM stannous chloride. Elemental analysis showed stannous as the depositing material within and on bacterial membranes and notably in the explosive vesicles. (**A**,**C**) are black-white micrographs. (**B**,**D**) were the signals from energy dispersive spectroscopic analysis. (**E**,**F**) are EDS spectrum peaks, with (**E**) showing particles in the matrix and (**F**) showing particles in the cell.

To determine whether the electron-dense spots within the outer and cytoplasmic membranes are stannous fluoride, we analyzed those electron-dense spots for elemental compositions in Figures 8A,C using EDS. As marked in the red cycle, elemental tin was detected in the electron dense spots within the bacteria membranes (Figures 8A,B). Tin(II), also known as stannous, is reacted with fluoride and chloride to form stannous fluoride and stannous chloride, respectively. In the high-resolution image (Figure 8C), three vesicles were observed in the upper region. The vesicle, marked by a yellow arrow, contained tin localized in the electron-dense spot (Figure 8D). Similarly, the two electron dense spots within the membranes and one right below the cytoplasmic membrane were stannous-positive. These results demonstrate that stannous aggregates formed within and around the bacterial membranes. Furthermore, these aggregates destabilized and fragmented bacterial membranes, which self-annealed to form explosive vesicles. Consequently, cellular organelles leaked out and bacteria broke into pieces.

## 4 Discussion

Among the oral microbiota, *Bacteroidetes* is one of the major phylae (containing *P. gingivalis* species) and *Prevotella* its largest genus (30). These anaerobic bacteria constitute a significant part of oral microbial communities. Oral *Prevotella* are known as anaerobic species and are observed in dental plaques from early life onwards, including pigmented *P. melaninogenica, P. nigrescens,* *P. pallens,* and some non-pigmented *Prevotella* species. Many *Prevotella* species contribute to oral inflammatory processes, being frequent findings in dysbiotic biofilms of periodontal diseases. Our findings relative to *P. pallens* are likely generalizable to other *Prevotella* species given their close genetic relationship.

*P. gingivalis* has been detected in high relative abundance in experimental gingivitis and found to be differentially abundant in chronic periodontitis compared to health (14). Considerable research has shown that *P. gingivalis* is a major etiologic factor contributing to chronic periodontitis (31). This black-pigmented bacterium produces a myriad of virulence factors that cause destruction to periodontal tissues either directly or indirectly by modulating the host inflammatory response (32–34). Antibodies to *P. gingivali*s can be detected in patients (35) and infection has been linked to Alzheimer's disease (36) and rheumatoid arthritis (37, 38).

**Figure 9:**
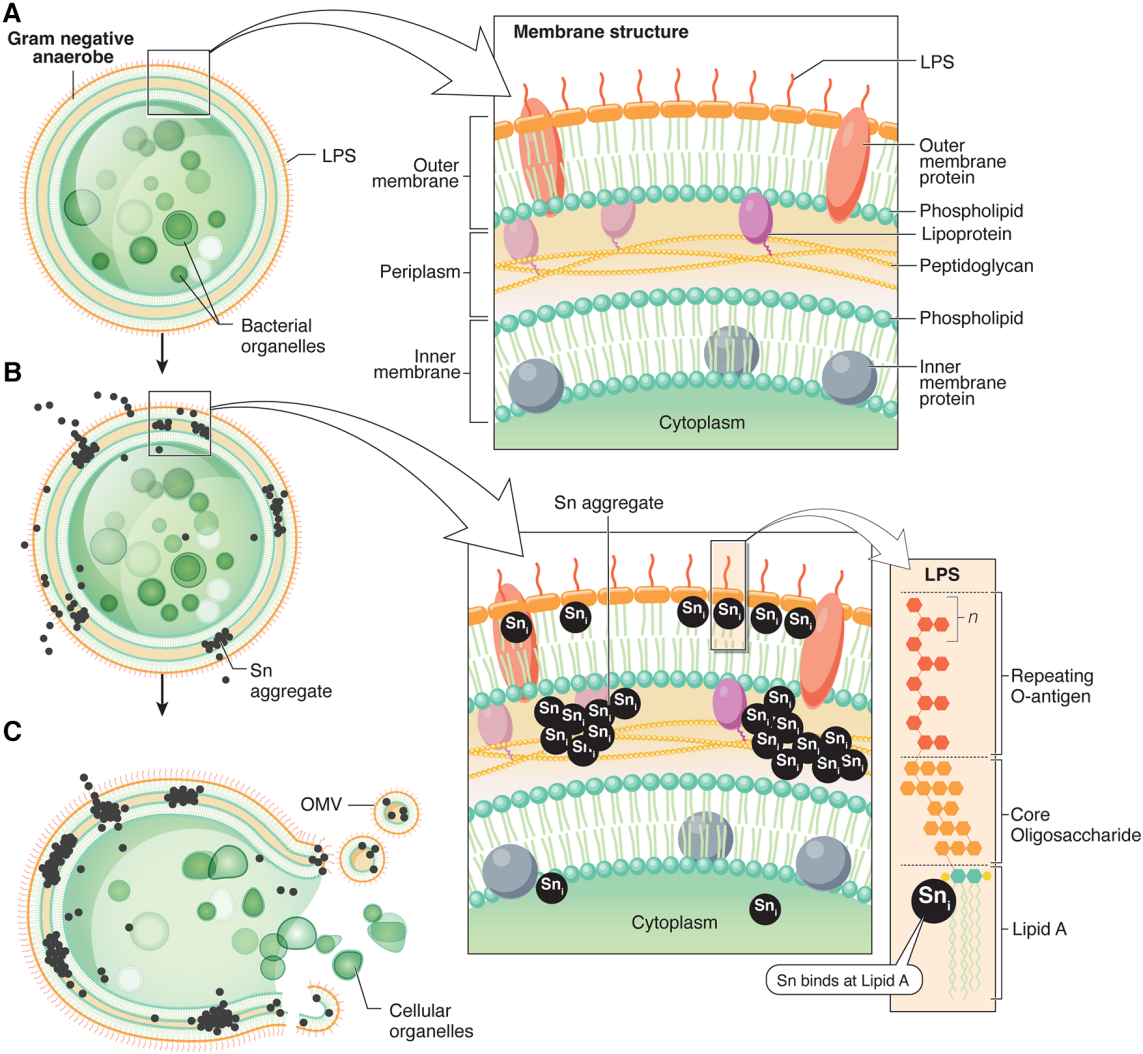
Schematic summary of the bactericidal mechanism of action for stannous. (**A**) Membrane structure of anaerobic Gram-negative bacteria *P. gingivalis*, adapted from Yoshimura et al. (39), and chemical ultrastructures of pathogen membrane components*.* (**B**) Proposed locations of stannous deposition apparent from TEM images in *P. pallens* and *P. gingivalis.* Stannous fluoride binds Lipid A in a one-to-one ratio. (**C**) Stannous aggregates lyse the cell membranes, forming disruptive membrane vesicles and releasing cellular contents.

The histomorphology of anaerobic bacteria like *P. pallens* and *P. gingivalis* includes a structure with both an inner and outer membrane, with both membranes being bilayers. The outer membrane typically includes a bilayer comprised of lipopolysaccharide on the surface with a layer of phospholipid underneath. The inner membrane is comprised of a bilayer of phospholipids. Transmembrane proteins are present in both internal and external membranes of the organisms. A peptidoglycan typically acts to bridge the two membranes. The structure as described by Yoshimura et al. (39) is illustrated in Figure 9A, along with the chemical structures of some of the membrane components.

The histomorphology observed in organisms cultured with stannous fluoride and stannous chloride in this study suggests that chemical precipitation and aggregation of insoluble tin salts is the primary reactivity responsible for bactericidal actions. It is known from prior studies (6) that stannous binds directly to lipopolysaccharides primarily at Lipid A (6, 7). Consideration of the detailed ultrastructure of the LPS from *P. gingivalis* suggests that an attractive site for Lipid A binding would be on terminal phosphate in the complex comprising Lipid A as described by Kamuda et al. (40). The Lipid A structure in the cell membranes extends into the bilayer beneath. Likewise, it is noteworthy that the area spanning the membranes is comprised of phospholipids with the polar phosphate moieties localized inside the bilayer between the membrane. The polar phosphate head groups of the inner membrane would be localized on the internal surface of the membrane. It is instructive that both regions of the cell membranes are rich in phosphate groups from Lipid A and phospholipids. Stannous routinely forms precipitates with phosphates (41). It is our conjecture that these groups contribute to stannous deposition in cell membranes of *P. pallens* and *P. gingivalis*. These ultrastructural observations suggest that stannous fluoride produces bactericidal actions on anaerobic pathogens by forming insoluble aggregates in the cell membranes. This creates a physical pressure in the membranes eventually producing their lysis. This mechanism is illustrated in Figures 9B,C. The explosive lysis of the cell membrane with stannous treatment (e.g., clearly shown in Figure 5) resembles lytic actions seen for membrane specific antibiotics with various organisms (42). This research demonstrates a statistically significant dose dependent suppression of *P. gingivalis* and *P. pallens* cell growth starting at 100–200 µM. Future investigations could build on these findings by evaluating total DNA released within the samples to see if it occurs in a dose-dependent manner that is positively correlated with bacterial growth inhibition.

The antibacterial activity of stannous fluoride has previously been the subject of several studies. Tsao et al. (43) observed stannous fluoride to be bactericidal to a variety of periodontal pathogens at concentrations below that which are used clinically. In a study demonstrating effects of stannous fluoride on gene expression of *S. mutans* and *Actinomyces*, MIC and MBC activities were observed at concentrations 10-fold less than those applied clinically (44). Mayhew and Brown (45) observed suppressed growth of *S. mutans* at concentrations as low as 75 ppm. Haraszthy et al. (46) examined antimicrobial activity of dentifrice dilutions on over 20 oral bacterial species. *Prevotella* and *P. gingivalis* species were inhibited at dentifrice diluents in media from 1.8–7.5 ppm. Weber et al. (47) observed bactericidal activities of stannous fluoride for both aerobic and anaerobic bacteria at diluted concentrations of stannous fluoride in dentifrice in time-kill experiments. Stannous fluoride exhibited bactericidal actions in time periods as low as two minutes of exposure.

Tinanoff and colleagues carried out extensive studies on stannous fluoride antimicrobial actions concentrating on actions on cariogenic organisms including *S. mutans* (17, 48, 49). These studies included histomorphological characterization of stannous fluoride interactions with *S. mutans*. For *S. mutans*, the observed antimicrobial properties were unique to stannous fluoride and involved intracellular retention of tin. The authors speculated that the intracellular concentration of stannous was associated with condensation into granules into these bacteria. The results in this study contrast with those observed for *S. mutans* as both *P. pallens* and *P. gingivalis* showed reactivity in the cell walls of these periodontal pathogens. The ultrastructure of the anaerobic pathogens differs from *S. mutans* in particular, including LPS and phospholipids in their cell membranes. *S. mutans* in contrast exhibit cell walls rich with a distinct polysaccharide composed of a poly-rhamnose core and glucose side chains.

The clinical efficacy of stannous fluoride for gingival health includes a number of complementary mechanisms including modulation of bacterial metabolism as well as direct suppression of virulence through binding of endotoxins. The bactericidal actions of stannous fluoride on periodontal pathogens shown here may complement these mechanisms to provide improvements in gingival health associated with dentifrices containing this ingredient. It is worth noting that stannous concentration in the subgingival crevicular fluid is above 200 μM at 30 min after brushing with stannous fluoride toothpaste (50). Our data demonstrates that low concentrations of stannous still have anti-microbial activities.

## 5 Conclusion

Our results elucidate a new mechanistic role for stannous fluoride in the cell membrane deposition, disruption, and destruction of *P. gingivalis* and *P. pallens* that leads to cell death. Stannous aggregation is apparent at concentrations as low as 25 mM of stannous fluoride, with suppression of growth curves starting at 100 mM. This mechanism likely plays an important role in the antibacterial and antigingivitis clinical efficacy of stannous fluoride.

## Data Availability

The raw data supporting the conclusions of this article will be made available by the authors, without undue reservation.
