# Supplementary material for: Stannous fluoride forms aggregates between outer and inner membranes leading to membrane rupture of Porphyromonas gingivalis and Prevotella pallens
Source: Front Oral Health. 2024 Jun 26;5:1427008. doi: 10.3389/froh.2024.1427008 (PMC11233731; doi:10.3389/froh.2024.1427008)
Supplement: Supplementary file 1 [file Datasheet1.docx]

Supplementary Material

# Supplementary Data – Methods

# SEM sample preparation and imaging: Bacterial cultures were centrifuged to collect the bacteria after 48 hours, and the bacterial pellets were placed in a fixative solution (2% glutaraldehyde in PBS buffer) immediately and stored at 40°C. The fixed samples were post-fixed with 1% OsO4 in PBS buffer overnight at 40°C. The samples were then dehydrated by gradually increasing the concentration (50%, 70%, 85%, 90%, 100%) with ddH2O for 3 h at room temperature. The dehydrated samples were deposited onto a filter paper and secured with silver paint on a sample mount. Additional sputter-coating with Au/Pd was performed in a Gatan Alto 2500 (Ametek, Berwyn, PA) for 60 seconds. High-resolution imaging was performed in a Hitachi S-4700 (Hitachi High-Tech, Hillsboro, OR) SEM with an accelerating voltage of 3 kV.

# MTGE-anaerobic enrichment broth, stannous fluoride 0.454% W/W toothpaste (Crest Pro-Health, Procter & Gamble, Cincinnati, OH), and pure water were acclimated under an anaerobic condition to remove residual oxygen. Toothpaste solution was freshly prepared right before the experiment. Toothpaste was weighed and dissolved in UPW water at 10% as stock solutions. The toothpaste stock was vortexed for 30 min in flasks to assure complete suspension of paste into water. Then the stock solution was diluted with MTGE enriched broth to culture bacteria (*P. pallens* ATCC 700821 and *P. gingivalis* ATCC 33277) under anaerobic conditions with a N2, CO2, H2 gas mixture ratio of 80:10:10 at 37°C for 48 hours in a 5 ml Falcon tube. Finally, the bacteria were harvested and processed for TEM observation.

# Supplementary Figures


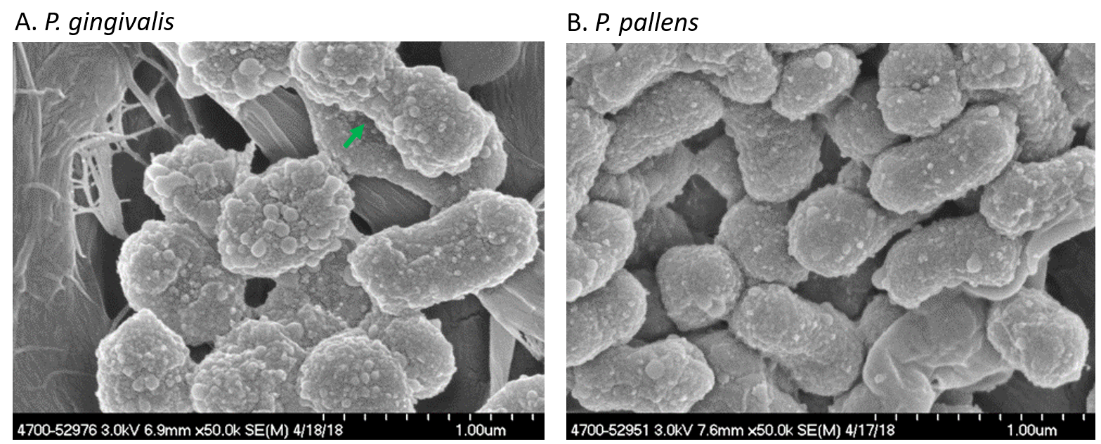


**Supplementary Figure 1.** Scanning electron microscopic imaging of *P. gingivalis (A)* and *P. pallens* (B) cultured in MTGE medium. Both *P. gingivalis* and *P. pallens* are short rod-shaped in broth culture. Small-sized outgrowths are numerous on the surface in culture. The outgrowths are smaller and fewer in *P. pallens* than in *P. gingivalis*. A bacterium was under binary fission as indicated by a green arrow.


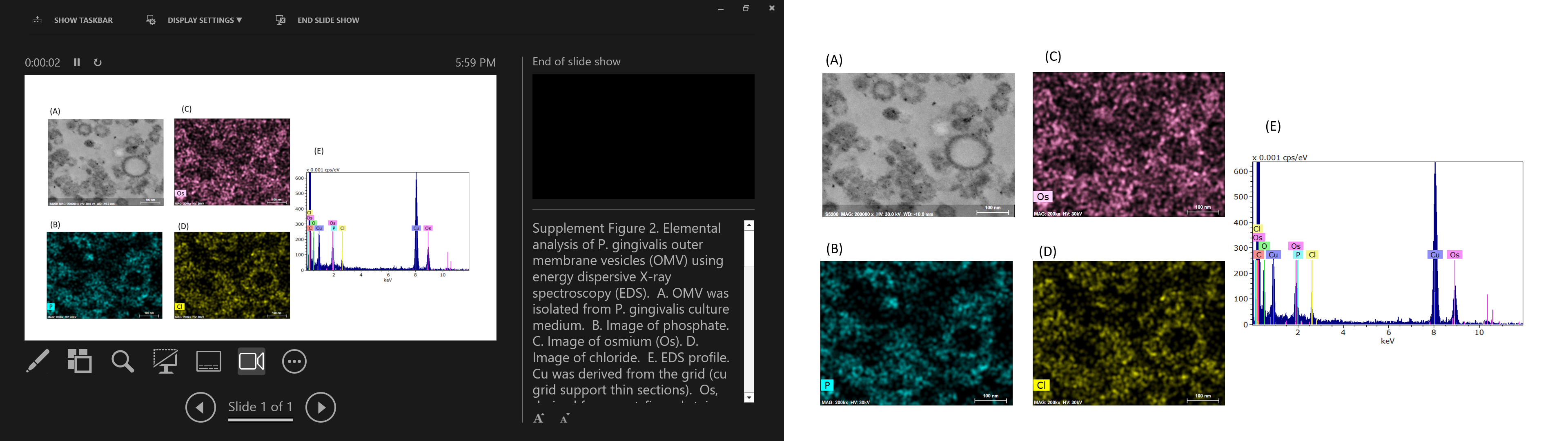


**Supplementary Figure 2.** Elemental analysis of *P. gingivalis* outer membrane vesicles (OMV) using energy dispersive X-ray spectroscopy (EDS). A. OMV was isolated from *P. gingivalis* culture medium. B. Image of phosphate. C. Image of osmium (Os). D. Image of chloride. E. EDS profile. Cu was derived from the grid (cu grid support thin sections). Os, derived from post-fix and stain, was the major metal associated with the OMV. Phosphate and chloride were minor components associated with the cells and were likely derived from buffer. Therefore, dark particles in samples, that were not treated with stannous fluoride or stannous chloride, were most likely associated with osmium indicated by significantly high atomic contrast.


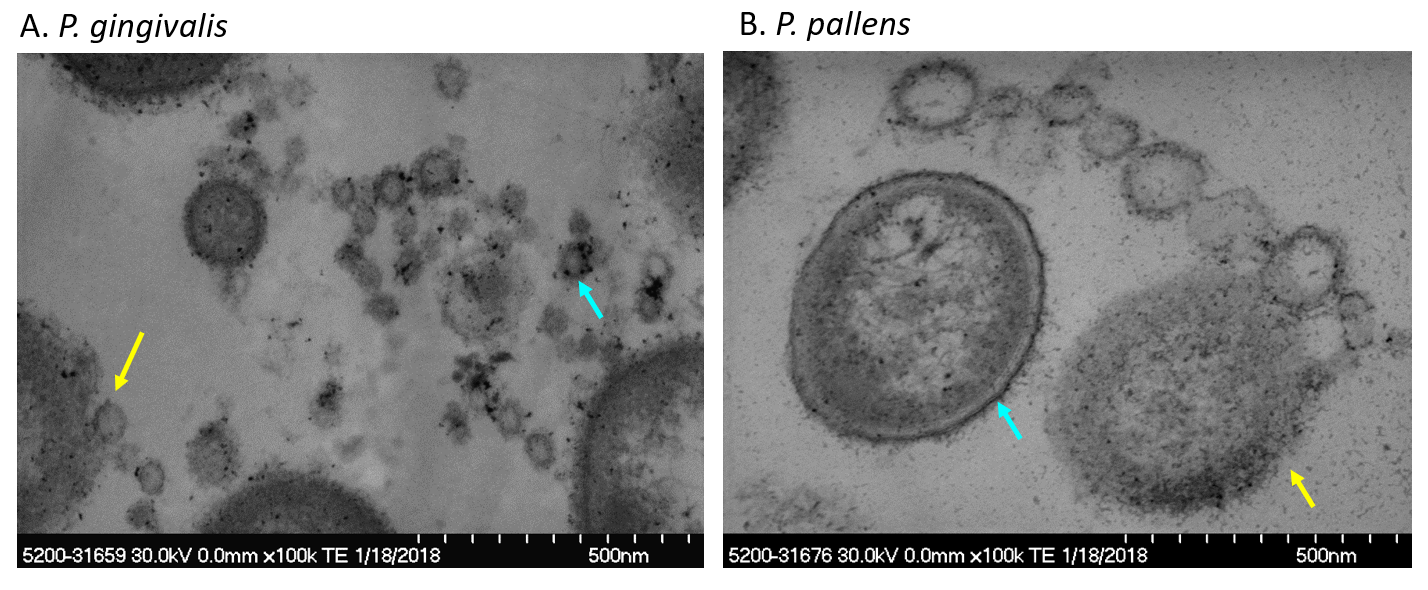


**Supplementary Figure** **3.** Micrographs of *P. gingivalis* and *P. pallens* cultured in MTGE medium with 0.1% Crest Pro-Health toothpaste solution. Crest Pro-Health toothpaste contains stannous fluoride at a concentration of 0.454% (1100 ppm fluoride), which is equivalent to 0.14% w/v fluoride ion. Numerous small vesicles were found near a *P. gingivalis* bacterium. Electron-dense spots were detected on the membranes of vesicles as marked by a yellow arrow. Electron-dense spots were also noted in the membranes of a *P. pallens* as indicated by turquoise arrow. A chain of vesicles was rolled out from the bacterium, marked by a yellow arrow.
